# ^18^F-NaF PET/CT and Extraordinary Involvement: Non-calcific Brain Involvement in a Prostate Cancer Case

**DOI:** 10.4274/mirt.galenos.2019.85547

**Published:** 2020-02-17

**Authors:** Ulku Korkmaz, Funda Ustun

**Affiliations:** 1 Trakya University Faculty of Medicine, Department of Nuclear Medicine, Edirne, Turkey

**Keywords:** NaF, PET, brain, metastasis

## Abstract

With the increase in the diagnosis of the cancer, the frequency of using imaging methods for diagnosis and for staging is also increased. Because of the complex structure of cancer and tumor behavior, the assessment methods have been updated and metabolic imaging has gained weight. The most popular of these techniques is hybrid positron emission tomography/computed tomography (PET/CT) systems. Prostate cancer is the second most common cancer in the world, is the fifth common type in cancer-related male deaths. Estimation of prognosis and treatment planning of the patients are based on the TNM classification. Bone metastasis is a prognostic factor of morbidity and mortality in prostate cancer. Sodium fluoride (NaF) PET/CT is a promising imaging modality in evaluation of skeletal system. This article will review the involvement of ^18^F-NaF in extra-osseous tissues in the prostate cancer and reveal the fundamental differences between ^18^F-NaF imaging and ^18^F-FDG imaging in these areas.

## Abstract (tr)

Kanser tanısının artmasıyla birlikte tanı ve evreleme için görüntüleme yöntemlerinin kullanım sıklığı da artmaktadır. Kanserin ve tümör davranışının karmaşık yapısından dolayı değerlendirme yöntemleri güncellenmiştir ve metabolik görüntüleme ağırlık kazanmıştır. Bu tekniklerin en popüler olanı hibrid pozitron emisyon tomografi/bilgisayarlı tomografi (PET/BT) sistemleridir. Prostat kanseri dünyada en sık görülen ikinci kanser ve erkeklerde kanserle ilgili ölüm nedenleri arasında beşinci en sık nedendir. Kemik metastazı, prostat kanserinde morbidite ve mortalite açısından prognostik bir faktördür. Sodyum florür (NaF) PET/BT, iskelet sisteminin değerlendirilmesinde umut verici bir görüntüleme yöntemidir. Bu makale ^18^F-NaF’nin prostat kanserinde kemik dışı dokulardaki tutulumunu gözden geçirerek bu alanlarda ^18^F-NaF görüntüleme ve ^18^F-FDG görüntülemenin temel farklılıklarını ortaya koyacaktır.

## Figures and Tables

**Figure 1:**
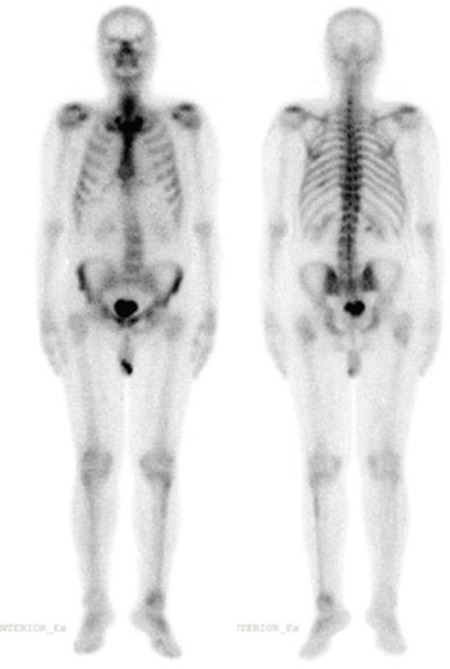
A 74-year-old male was diagnosed as having Gleason 3+7=(10) prostate adenocarcinoma and underwent technetium (Tc) Tc-99m methylene diphosphonate (MDP) whole body bone scintigraphy (WBBS) for staging. There was suspicious metastatic involvement in right 9^th^ rib, right acetabulum and left fibula/proximal tibia in WBBS and sodium fluoride (NaF) positron emission tomography/computed tomography (PET/CT) scan was done for further evaluation.

**Figures 2, 3:**
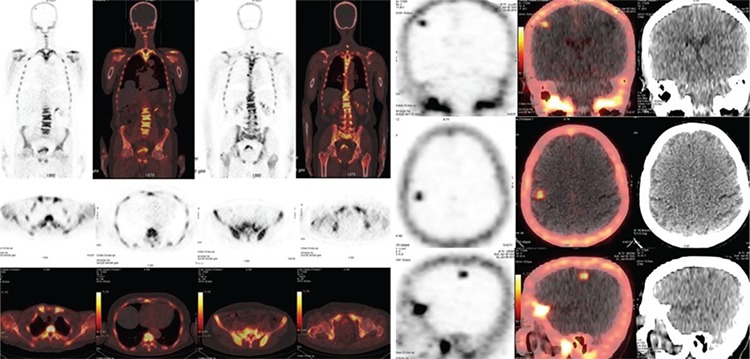
NaF PET/CT images showed widespread involvement in the skeletal system, including MDP avid lesions. Also NaF involvement was observed in the right parietooccipital field in the cranium (SUV_max_: 4.8), with no calcification or identifiable lession in the unenhanced CT counterpart. The patient was evaluated in neurology and radiation oncology clinics and radiotherapy and chemotherapy were initiated for extracranial metastases. The patient was followed up with androgen receptor blocker and did not receive additional treatment for intracranial mass. After three years of follow up for ekstracranial disease, he was admitted to the emergency room with an epileptic seizure due to intracranial mass on June 2018. The mass was accepted as a metastatic lesion and he is still receiving a treatment for this. (SUV_max_: Maximum standardized uptake value) Bone metastasis is a prognostic factor in prostate cancer and the ratio was reported as 70% in autopsy series (1). Recently, the assessment methods have been updated and hybrid metabolic imaging (PET/CT, PET/MR) has gained weight. The most widely used PET radiopharmaceutical is ^18^F-FDG. This molecule is a very good metabolic marker for soft tissue and bone marrow, however, it does not reach intended sensitivity and specificity to be accepted as a classical agent for bone imaging, especially in cases with involvement of the cortical bone. While the phosphate groups marked with Tc-99m are used as main method for the detection of bone metastases, technical developments have allowed the spread of ^18^F-NaF for this purpose (2). ^18^F-NaF is retained by mineralized bone tissue in proportionally with the osteoblastic activity (3). Tc-99m phosphanats are mostly involved in osteoblastic metastases and fluorodeoxyglucose PET/CT is more associated with bone marrow involvement, ^18^F-NaF PET/CT shows better involvement in both sclerotic and lytic metastases (4). Whereas ^18^F-NaF shows high affinity to osseuos tissue, it is not retained by normal brain tissue and facilitates the seen of bone structures (4). Almost all of extra-osseous ^18^F-NaF involvements are in brain tissue (5). Physiologically, ^18^F-NaF can not pass the blood-brain barrier. However, if the blood brain barrier is broken down for a reason, the metastatic tumor cells can settle here. The involvement of bone seeking agents in brain metastases, is not just because of deterioration of the blood brain barrier, but also because of the metabolic uptake mechanisms of tumor cells (6). For example, fibril structures and amyloid foci have been reported to exhibit affinity for calcium, physiologically (7). Furthermore, Ca-L, an ion channel, has been shown to be present in pancreatic cancer cells and is effective in tumor pathogenesis (8). A similar mechanism could also be possible for sodium mediated ion channels and ^18^F-NaF. As a result, brain metastasis may be detected incidentally in ^18^F-NaF-images and that unexpected involvement should be carefully evaluated before it is considered as an artifact. This precise approach is necessary to prevent the false staging of the patient.
